# Revisiting bilateral bony orbital volumes comparison using 3D reconstruction in Korean adults: a reference study for orbital wall reconstruction, 3D printing, and navigation by mirroring

**DOI:** 10.1186/s12893-023-02268-0

**Published:** 2023-11-17

**Authors:** Hyung Min Hahn, Yeon Kyo Jung, Il Jae Lee, Hyoseob Lim

**Affiliations:** Department of Plastic and Reconstructive Surgery, Ajou University School of Medicine, 164 World Cup-Ro, Yeongtong-Gu, Suwon-Si, Gyeonggi-Do 16499 Republic of Korea

**Keywords:** Orbit, Age groups, Reconstructive surgical procedure, Image processing, Computer-assisted, Orbital implants, Mirroring

## Abstract

**Background:**

Orbital wall fractures can result in changes to the bony orbital volume and soft tissue. Restoring the bony orbital and intraconal fat volumes is crucial to prevent posttraumatic enophthalmos and hypoglobus. We aimed to establish an evidence-based medical reference point for “mirroring” in orbital wall reconstruction, which incorporates three-dimensional (3D)-printing and navigation-assisted surgery, by comparing bilateral bony orbital volumes.

**Methods:**

We retrospectively analyzed the data obtained from 100 Korean adults who did not have orbital wall fractures, categorized by age groups. The AVIEW Research software (Coreline Soft Inc., Seoul, South Korea) was used to generate 3D reformations of the bony orbital cavity, and bony orbital volumes were automatically calculated after selecting the region of interest on consecutive computed tomography slices.

**Results:**

The mean left and right orbital volume of males in their 20 s was 24.67 ± 2.58 mL and 24.70 ± 2.59 mL, respectively, with no significant difference in size (*p* = 0.98) and Pearson’s correlation coefficient of 0.977 (*p* < 0.001). No significant differences were found in orbital volumes in other age groups without fractures or in patients with nasal bone fractures (*p* = 0.84, Pearson’s correlation coefficient 0.970, *p* < 0.001). The interclass correlation coefficients (2,1) for inter- and intrarater reliability were 0.97 (*p* < 0.001) and 0.99 (*p* < 0.001), respectively.

**Conclusions:**

No significant differences were found in the bilateral bony orbital volumes among males of any age. Thus, the uninjured orbit can be used as a volumetric reference point for the contralateral injured orbit during orbital wall reconstruction.

## Background

Orbital wall fractures can result in changes to the bony orbital volume and soft tissue. An increase in the orbital volume is strongly correlated with the degree of enophthalmos [1, 2], whereas the intraconal fat volume is an important determinant of eyeball projection, the reduction of which can result in complications, including posttraumatic enophthalmos and hypoglobus. As such, restoration of the bony orbital and intraconal fat volumes is important for preventing posttraumatic enophthalmos and hypoglobus. Several methods are available to recover the intraconal fat volume, including retrobulbar fat injection; however, these are associated with potential complications [3]. Therefore, bony orbital wall reconstruction is sometimes applied to prevent complications such as orbital wall fracture and orbital soft tissue herniation. For this procedure, manual, patient-specific, and autologous bone transplantation may be used.

During orbital wall reconstruction, thorough defect assessment and accurate restoration are indispensable to prevent complications, including enophthalmos, diplopia, and cheek or nasal numbness [4]. Accordingly, surgical management using three dimensional (3D)-navigation can be a safe, precise, and efficacious method, resulting in good functional and aesthetic outcomes for orbital wall fractures [5]. Recent advances in 3D printing have allowed the production of patient-specific implants for orbital defects, facilitating improvements in reconstructive outcomes [6]. Moreover, “mirroring” is crucial for utilizing 3D-navigation and producing patient-specific implants through 3D printing. This practice is empirically based on establishing symmetry with the unaffected orbit to decide on the normal orbital volume [7]. Symmetry of bony orbital volumes is gaining importance owing to the emergence of reconstruction procedures using 3D-navigation or pre-molded patient-specific implants [6, 8–10]; however, previous studies reported conflicting results regarding the symmetry between bilateral orbital volumes. Lieger et al. [11] observed that the difference between bony orbital volumes was small but significant. In contrast, Shyu et al. [12] and Kim et al. [13] showed that the discrepancy between bilateral orbital volumes was not significant. Therefore, this study aimed to provide a reference point for “mirroring” in orbital wall reconstruction through a review of ophthalmic symmetry, stratified by age group and sex.

## Methods

### Study participants

We retrospectively analyzed the data collected from 100 Korean adults between January and December 2022. In total, we included 10 males and 10 females in each age group (20 s, 30 s, 40 s, 50 s, and 60 s), who experienced facial injuries without orbital wall fractures. Further, this retrospective study led to the inclusion of patients diagnosed with nasal bone fractures. Accordingly, our investigation focused on determining the impact of nasal bone fractures on orbital volume among individuals who solely had nasal bone fractures or did not have any facial fractures. This study was approved by the appropriate institutional review board of Ajou Medical Center (AJOUIRB-DB-2023–018) and adhered to the Declaration of Helsinki on Medical Protocol and Ethics. Data were collected through a patient chart review and included facial computed tomography (CT). CT was performed to evaluate facial injuries, specifically facial fractures, in patients who visited our hospital’s emergency room, trauma center, and plastic surgery clinic. Patients who had no facial bone fracture and were treated for simple nasal bone fractures with intact orbital walls were included. Patients were excluded with intraocular tumors, thyroid diseases, congenital or acquired ophthalmic diseases, previous orbital or eyeball surgery, orbital wall fracture, and neighboring fracture such as zygomaticomaxillary complex fracture, LeFort II/III fracture, and naso-orbito-ethmoidal fracture.

### Measurements of bony orbital volume

CT images were acquired using a 16-section multidetector row CT scanner (SOMATOM Sensation 16, Siemens Medical Solutions, Erlangen, Germany) and 64-section multidetector CT scanner (Brilliance 64, Philips Medical Systems, Best, Netherlands). The imaging parameters were: voltage, 120 kV; current, 250 mA; matrix, 300 × 2000; maximal scanning time, 10 s; and section thickness, 1 mm.

Corrected sagittal, coronal, and axial images were exported to the Digital Imaging and Communications in Medicine (DICOM) file format and viewed using a picture archiving and communication system (PiView STAR; INFINITT, Seoul, South Korea).

**Fig. 1:**
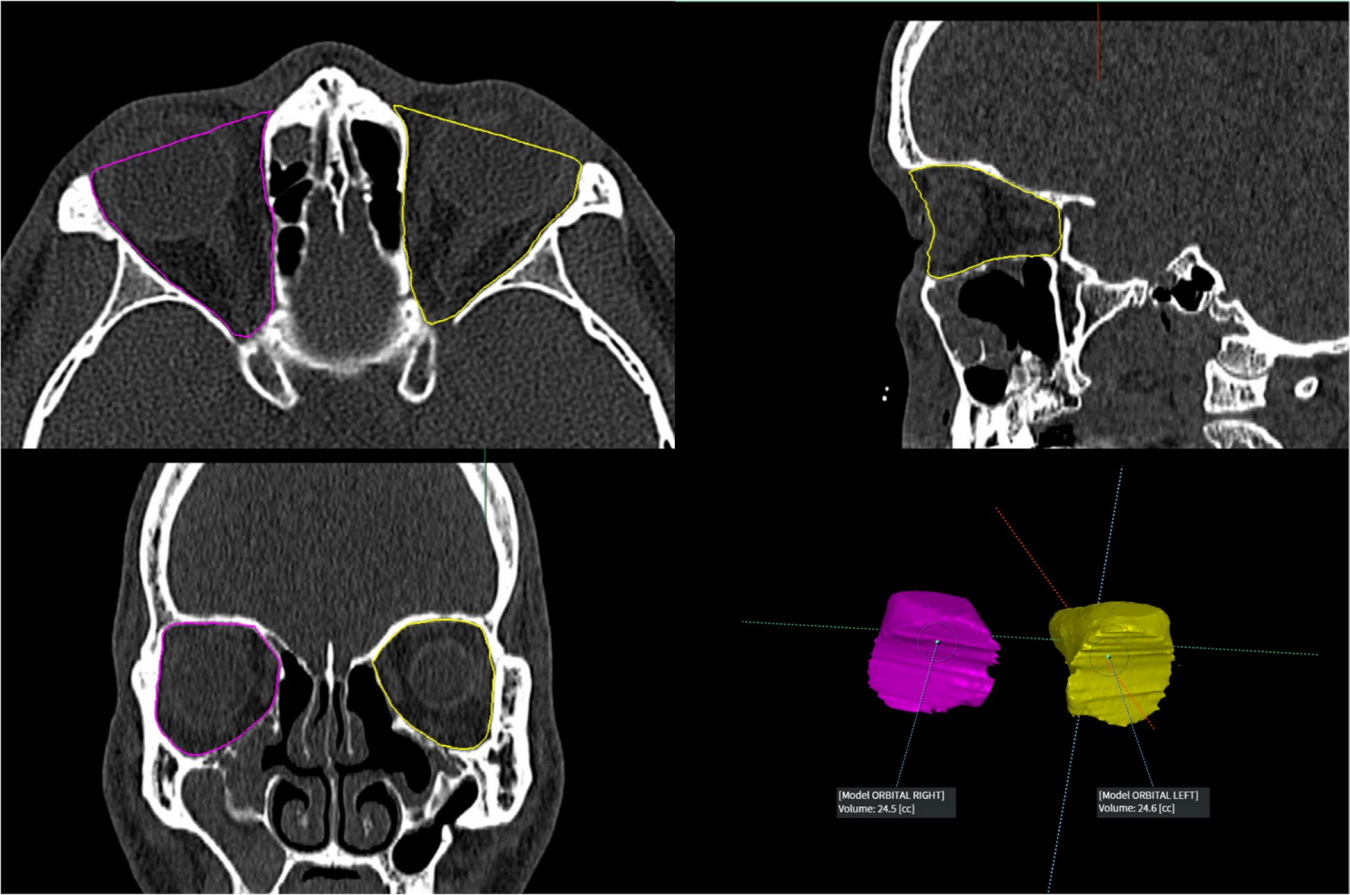
Overview of three-dimensional (3D) reformation of the bony orbital cavity and orbital volume measurements

AVIEW Research software (Coreline Soft Inc., Seoul, South Korea) was used to generate 3D reformations of the bony orbital cavity. To define a region of interest (ROI), landmarks were placed on the facial bone. These landmarks included the nasal process of the frontal bone for the superomedial orbital rim, anterior lacrimal crest for the inferomedial orbital rim, frontal process of the zygoma for the lateral orbital rim, and superior/inferior orbital rim [12, 14]. The nasolacrimal duct and optic canal were excluded from the measurements (Fig. 1). The bony orbital volumes were calculated after selecting the ROI on all the consecutive CT slices. Volume was obtained using the following formula: volume = (number of voxels) × (size of one voxel), where the size of one voxel = pixel spacing X (mm) × pixel spacing Y (mm) × slice interval (mm).

### Reliability assessments of orbital volume data

Interclass correlation coefficients (ICCs) were calculated to assess the reliability of the orbital volume measurement method. Rater 1 measured the orbital volume of all 100 patients (200 orbits) and re-measured 40 right orbital volumes 1 month later to determine the intrarater reliability. To assess interrater reliability, Rater 2 measured the same 40 right orbital volumes and the resulting data were then compared with the data measured by Rater 1. The total right orbital volume was measured in four males and four females aged 20–60 years, resulting in a total of 40 right orbital volumes. The basic assumptions of the ICC needed to meet the criteria of continuity and normality. The variables measured by all raters were continuous and normally distributed.

### Statistical analysis

An unpaired t-test was applied to analyze the differences between the left and right orbital volumes based on age and sex. The Pearson’s correlation coefficient test was used to confirm the correlation between the bilateral orbital volumes for all patients. An interclass correlation coefficient test was used to define the inter- and intrarater reliability. Statistical significance was set at *p* < 0.05. All statistical analyses were performed using SPSS 29.0.1.0 software (IBM Corporation, Armonk, New York, USA), and all graphs were generated using GraphPad Prism version 9.5.1 (733) for Windows (GraphPad Software, San Diego, California, USA).

## Results

### Comparison of left and right orbital volumes in males by age groups

The mean left and right orbital volume in males in their 20 s were 24.67 ± 2.58 mL and 24.70 ± 2.59 mL, respectively, with no significant difference (*p* = 0.98) and Pearson’s correlation coefficient of 0.977 (*p* < 0.001). Thus, the left and right orbital volumes had a strong positive correlation. Similarly, no significant differences were observed in the orbital volumes of the remaining age groups (Tables 1 and 2).

**Table 1:** Results of the independent-samples t-test for left and right bony orbital volumes by sex and age group

Age group (*n* = 10)	Left (Mean ± SD) (mL)	Right (Mean ± SD) (mL)	*p*-value
20 s male	24.67 ± 2.58	24.70 ± 2.59	0.98
20 s female	22.16 ± 1.60	22.36 ± 1.78	0.80
30 s male	26.63 ± 2.14	26.66 ± 2.33	0.98
30 s female	23.74 ± 1.95	23.70 ± 1.98	0.96
40 s male	27.76 ± 1.74	27.84 ± 2.11	0.93
40 s female	25.79 ± 1.05	25.84 ± 1.11	0.92
50 s male	28.18 ± 2.46	28.39 ± 2.27	0.85
50 s female	24.50 ± 2.04	25.06 ± 2.01	0.54
60 s male	26.59 ± 3.12	26.60 ± 3.06	0.99
60 s female	23.46 ± 1.86	23.81 ± 2.21	0.71
All ages (*N* = 100)	25.35 ± 2.76	25.50 ± 2.79	0.71

*SD* standard deviation

**Table 2:** Results of the Pearson’s correlation coefficient test for left and right bony orbital volumes

Age group (*n* = 10)	*R* value	*p*-value
20 s male	0.977	< 0.001
20 s female	0.961	< 0.001
30 s male	0.957	< 0.001
30 s female	0.967	< 0.001
40 s male	0.968	< 0.001
40 s female	0.832	0.003
50 s male	0.983	< 0.001
50 s female	0.922	< 0.001
60 s male	0.922	< 0.001
60 s female	0.955	< 0.001
All ages (*N* = 100)	0.969	< 0.001

### Comparison of orbital volumes by sex

The mean left orbital volume in males was 26.77 ± 2.65 mL, whereas that in females was 23.93 ± 2.06 mL (*p* = 0.08; Pearson’s correlation coefficient, 0.111 [*p* = 0.44]). The mean right orbital volume in males was 26.84 ± 2.71 mL, and that in females was 24.15 ± 2.16 mL (*p* = 0.10; Pearson’s correlation coefficient, 0.076 [*p* = 0.60]). Finally, the mean bilateral orbital volume in males was 26.77 ± 2.64 mL, and that in females was 24.04 ± 2.10 mL (*p* = 0.02; Pearson’s correlation coefficient, 0.094 [*p* = 0.36]).

### Comparison of left and right orbital volumes in patients with nasal bone fracture

Facial CT performed to evaluate facial injury revealed that 75 patients had nasal bone fractures, while the remaining 25 patients had no facial bone fractures. The mean left and right orbital volume of patients with nasal bone fracture were 25.68 ± 2.86 mL and 25.78 ± 2.93 mL, respectively. The difference between the left and right volumes was not statistically significant (*p* = 0.84), and the Pearson’s correlation coefficient was 0.970 (*p* < 0.001).

### ICC for inter- and intrarater reliability

The variables measured by Raters 1, 2, and 1 (after 1 month) were continuous and followed a normal distribution, thereby satisfying basic assumptions of the ICC. In interrater reliability evaluation between Raters 1 and 2, the ICC (2,1) was 0.97 (*p* < 0.001); therefore, the consistency of volume measurements by Raters 1 and 2 was 97%. Assessment of the intrarater reliability revealed that, for Rater 1, the ICC (2, 1) was 0.99 (*p* < 0.001), indicating a 99% consistency in volume measurements by Rater 1 at different time point.

## Discussion

Our study aimed to provide an evidence-based medical reference point for “mirroring” of orbital volumes in orbital wall reconstruction by comparing volumes of the left and right orbits based on sex and age group (20 s to 60 s). According to Lieger et al., there was a statistically significant but quantitatively small difference in the bilateral bony orbital volume. The largest asymmetry in their data set not found in our data set was for a patient who displayed a 10% difference in bony orbital volume. We think this distinction appears to have led to a somewhat different conclusion than what our study found.

Subsequently, this study showed sufficient reliability for volume measurements through intra- and interrater reliability measurements. Overall, we found no statistically significant differences in the average left and right orbital volumes in any age group, whereas correlation analysis showed a strong positive correlation between the left and right orbital volumes. This finding suggests that the orbital volume was consistent between the two sides, implying that the uninjured orbit can serve as a volumetric reference for the injured orbit on the opposite side.

In patients with unilateral orbital wall fracture, there is a good chance that the defect size and displacement are minimal if there is no difference in the bilateral bony orbital volume. At this point, orbital reconstruction can be carried out by estimating the pre-fracture line of the affected orbit based on the intact orbit, provided that patients with severe congenital or acquired facial asymmetry are excluded. In the case of bilateral orbital wall fracture, a pre-fracture line can be expected by overlapping the intact part of the opposite side if the fractured part is not the same.

CT evaluation was performed in patients who visited the hospital with facial injuries. Those with only nasal bone fractures or no facial fractures were selected for inclusion. As the zygomaticomaxillary complex accounts for a large portion of the orbital wall, fracture of this structure affects the bony orbital volume. However, the nasal bone does not occupy an anatomical portion of the orbital wall. We assumed that nasal bone fractures, as opposed to naso-orbito-ethmoidal fractures, would not cause a change in orbital volume; however, the limitations of the retrospective study led to the inclusion of patients diagnosed with nasal bone fractures. To confirm this assumption, we examined whether nasal bone fractures affected orbital volume. Our findings revealed no difference in orbital volume between patients with and without nasal bone fractures. Thus, we confirmed that nasal bone fractures did not affect the bony orbital volume.

In this study, we identified a statistically significant difference in orbital volume between male and female patients; our results showed that the bony orbital volume was larger in males. Prior studies have revealed that the orbital tissue volume is larger in males than in females, and that orbital tissue volume increases as body mass index increases. Orbital tissue and bony orbital volumes are not necessarily correlated; however, on average, the body mass of males is larger than that of females [15, 16].

To enhance the accuracy of our evaluation, we assessed the intra- and interrater reliability of our method, which revealed an agreement rate of 97% and 99%, respectively. Therefore, the bony orbital volume and other measurements used in this study were considered reliable.

**Fig. 2:**
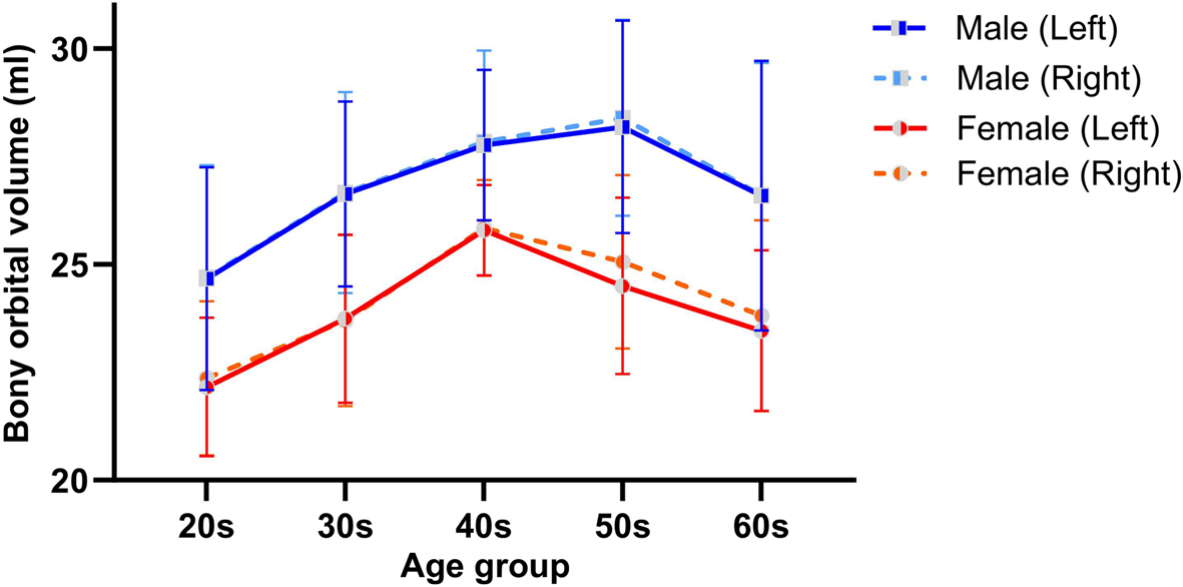
Bilateral bony orbital volumes by age group and sex

The bony orbital volume changes with age owing to changes in the orbital soft tissue. Although this study did not focus on longitudinal changes, we found that the bony orbital volume initially increased with age, before reaching a peak and then decreased again. In males, the bony orbital volume increased up to the 50 s age group and then decreased in the 60 s age group, whereas in females, the bony orbital volume increased up to the 40 s age group and then decreased in the 50 s and 60 s age groups (Fig. 2). Thus, these results showed that the bony orbital volume is linked to patient age. Further research with more patient groups and a more specific longitudinal design is needed to confirm these results [15–17].

The period of rapid orbital volume growth concludes for boys at approximately the age of 15 and for girls at approximately the age of 11 years [18]. Therefore, patients younger than 20 years were excluded, and only adult patients were included in this study.

## Conclusions

The left and right bony orbital volumes in adult patients in their 20 s to 60 s had no differences. Thus, we suggest that the uninjured orbit can be used as a reliable volumetric reference point for orbital wall reconstruction of the opposite injured orbit. On average, the bony orbital volume was larger in males than in females, and showed a changing trend with age, with volumes peaking in males in their 50 s and females in their 40 s.

## Data Availability

The data presented in this study are available on request from the corresponding author.

## Abbreviations

3D: Three-dimensional
CT: Computed tomography
DICOM: Digital Imaging and Communications in Medicine
ICC: Interclass correlation coefficients
ROI: Region of interest

## References

[1] Fan X, Li J, Zhu J, Zhang D (2003). Computer-assisted orbital volume measurement in the surgical correction of late enophthalmos caused by blowout fractures. Ophthalmic Plast Reconstr Surg.

[2] Ahn HB, Ryu WY, Yoo KW, Park WC, Rho SH, Lee JH (2008). Prediction of enophthalmos by computer-based volume measurement of orbital fractures in a Korean population. Ophthalmic Plast Reconstr Surg.

[3] DeSerres JJ, Budning A, Antonyshyn OM (2022). Current management of late posttraumatic enophthalmos. Plast Reconstr Surg.

[4] Cole P, Boyd V, Banerji S, Hollier LH (2007). Comprehensive management of orbital fractures. Plast Reconstr Surg.

[5] Yang JR, Liao HT (2019). Functional and aesthetic outcome of extensive orbital floor and medial wall fracture via navigation and endoscope-assisted reconstruction. Ann Plast Surg.

[6] Gander T, Essig H, Metzler P, Lindhorst D, Dubois L, Rücker M (2015). Patient specific implants (PSI) in reconstruction of orbital floor and wall fractures. J Craniomaxillofac Surg.

[7] Yu H, Shen G, Wang X, Zhang S (2010). Navigation-guided reduction and orbital floor reconstruction in the treatment of zygomatic-orbital-maxillary complex fractures. J Oral Maxillofac Surg.

[8] Kotecha S, Ferro A, Harrison P, Fan K (2023). Orbital reconstruction: a systematic review and meta-analysis evaluating the role of patient-specific implants. Oral Maxillofac Surg.

[9] Shin HS, Kim SY, Cha HG, Han BL, Nam SM (2016). Real time navigation-assisted orbital wall reconstruction in blowout fractures. J Craniofac Surg.

[10] Dubois L, Schreurs R, Jansen J, Maal TJ, Essig H, Gooris PJ (2015). Predictability in orbital reconstruction: a human cadaver study. Part II: Navigation-assisted orbital reconstruction. J Craniomaxillofac Surg.

[11] Lieger O, Schaub M, Taghizadeh E, Büchler P (2019). How symmetrical are bony orbits in humans?. J Oral Maxillofac Surg.

[12] Shyu VB, Hsu CE, Chen CH, Chen CT (2015). 3D-assisted quantitative assessment of orbital volume using an open-source software platform in a Taiwanese population. PLoS One.

[13] Kim SP, Lee BY, Lee SJ, Choi MH, Yeon HW, Park JY (2012). A study on orbital volume of Korean people in their 20s or 40s. Ophthalmic Res.

[14] Du Y, Lu BY, Chen J, He JF (2019). Measurement of the orbital soft tissue volume in Chinese adults based on three-dimensional CT reconstruction. J Ophthalmol.

[15] Yoo JH, Lee YH, Lee H, Kim JW, Chang M, Park M (2013). Correlation between orbital volume, body mass index, and eyeball position in healthy East Asians. J Craniofac Surg.

[16] Chon B, Zhang KR, Hwang CJ, Perry JD (2020). Longitudinal changes in adult bony orbital volume. Ophthalmic Plast Reconstr Surg.

[17] Kahn DM, Shaw RB (2008). Aging of the bony orbit: a three-dimensional computed tomographic study. Aesthet Surg J.

[18] Furuta M (2001). Measurement of orbital volume by computed tomography: especially on the growth of the orbit. Jpn J Ophthalmol.

